# 

**Published:** 1910-10

**Authors:** 


### CONTENTS.

ORIGINAL COMMUNICATIONS—
Some Diagnostic Points of Mental Diseases and Why the Commitment Pro ceedings of the Insane of Georgia Should be Entirely Revised. By J. Cheston King, A. B., M. D., Atanta, Ga. .......... 323
A Review of the Reports on the use Ehrlichs New Remedy for Syphilis. By Edgar G. Ballinger, M. D., Atlanta, Ga. .......... 335
Pellagra. By Thos. F. Taylor, Tuskegee, Ala. .......... 340
Report of Case of Pellagra, By Dr. A. D. McLain. Salem, Ala. .. .. 346
Discussion on the Papers of Drs. Taylor and McLain. By Dr. B. L. Wyman, Birmingham Ala. .......... 348
Christian Science "The Religo-Medical Masquerade." By Henry R. Slack, Ph. M., M. D. .......... 351
Intestinal Excessive Activity. Physiological Consideration. By G. M. Saleba, M. D., D. D. S., Roanoke, Ala. .......... 360
Discussions on the Paper of Dr. G. M. Saliba, Roanoke, Ala. "Intestinal Excessive Activity Physiological Consideration." .......... 367
A Case Pulmonary Tuberculosis Treated by Artificial Phemothorax. By Mary E. Lapham, M. D. Highlands Camp Sanatorium, Highlands, N. C. .......... 369
EDITORIALS .......... 371
NEWS AND NOTES .......... 373
Proceedings of The American Proctological Society. .......... 377

